# Correlation analysis of norepinephrine dose on enteral nutrition tolerance and prognosis in patients with septic shock

**DOI:** 10.1186/s12879-023-08366-x

**Published:** 2023-06-08

**Authors:** Fan Qi, Guangqing Huang, Hunian Li, Xu Zhao, Jie Liu

**Affiliations:** 1 Jinzhou Medical University, Jinzhou, China; 2 Emergency and Critical Care Center, Renmin Hospital, Hubei University of Medicine, Shiyan, Hubei 442000 China; 3 Maternal and Child Health Care Hospital Affiliated to Hubei University of Medicine, Shiyan, 442000 China

**Keywords:** Septic shock, Enternal nutrition, Norepinephrine, Hemodynamics, Intensive care unit

## Abstract

**Background:**

To explore correlation between the dose of norepinephrine and the timing of starting enteral nutrition in septic shock (SS) patients.

**Methods:**

Totally 150 SS patients treated with enteral nutrition (EN) in Shiyan People’s Hospital from Dece20 to July 2022 were included in this retrospective analysis. Patients were divided into tolerance group (n = 97) and intolerance group (n = 53) according to whether EN was tolerated or not. The study indexes include baseline characteristics [gender, age, weight, body mass index (BMI), scores of acute physiology and chronic health evaluation II system (APACHE II), comorbidity, time in-hospital, prognosis], clinical indexes [mean arterial pressure (MAP), time of mechanical ventilation (MV), norepinephrine dose at the time of starting EN, using of sedative drug, gastrointestinal motility drugs and cardiotonic drugs], EN indexes (timing of starting EN, speed of EN infusion, calorie of EN per day, EN target percent), and gastrointestinal intolerance index [residual gastric volume > 250 ml, vomiting, aspiration, gastrointestinal bleeding, blood lactic acid (BLA)]. Student-t test and Mann-Whitney test were used for test of measurement data. Chi-square test and fisher exact test were used for comparison of categorical data.

**Results:**

There were 51 (52.58%) male and 46 (47.42%) female patients with a median age of 66.4 ± 12.8 years old in tolerance group. There were 29 (54.72%) male and 24 (45.28%) female patients with a median age 67.3 ± 12.5 years old in intolerance group. The weight and BMI were significantly higher in intolerance group than those of tolerance group (both P < 0.001). There was no significant difference of comorbidity rate between two groups (all P > 0.05). Before the overlapping time of EN and norepinephrine, there were significantly more patients receiving gastrointestinal motility drugs in intolerance group compared with tolerance group (58.49% vs. 20.62%, P < 0.001). Patients in tolerance group had significantly less residual volume in gastric than that of intolerance group (188.00 ± 52.32 vs. 247.83 ± 34.95, P < 0.001). The rate of residual volume in gastric > 250ml (9.28% vs. 37.74%, P < 0.001), vomiting (15.46% vs. 35.85%, P = 0.004) and aspiration(16.49% vs. 33.96%, P = 0.018) were significantly lower in tolerance group than those of intolerance group. The BLA in tolerance group was significantly lower than that of intolerance group (1.84 ± 0.63 vs. 2.90 ± 1.5 3mmol/L,P < 0.001). There were significantly more patients with increased BLA (75.47% vs. 30.93%, P < 0.001) and > 2mmol BLA rising (43.40% vs. 8.25%, P < 0.001) in intolerance group than those of tolerance group. Patients in tolerance group had significantly lower time of starting EN (40.97 ± 9.53 vs. 49.85 ± 11.61 h, P < 0.001), dose of NE(0.23 ± 0.07 vs. 0.28 ± 0.10 ug/kg/min, P = 0.049), mortality in hospital (18.56% vs. 49.06%, P < 0.001) and mortality in ICU (16.49% vs. 37.74%, P < 0.001) compared with intolerance group. The EN target percent (92.78% vs. 56.60%, P < 0.001) and calorie of EN during overlapping period (20.22 ± 5.99 vs. 16.21 ± 2.52 kcal/kg/day, P < 0.001) in tolerance group were significantly higher than those of intolerance group.

**Conclusions:**

SS patients should be comprehensively evaluated according to their condition. Obese patients are more prone to EN intolerance, and those who can tolerate EN should be implemented as soon as possible. The use dose of NE is significantly related to EN tolerance. When the use dose is low, EN tolerance is greater.

## Introduction

Septic shock (SS) is the most serious septicemia and the main cause of death in intensive care unit (ICU). It is emphasized that SS patients are not only with severe circulatory failure, but also combined with metabolism disorder and organ damage induced by infections, which results in higher death risks than spesis alone [1]. Sepsis is manifested as shock, systemic inflammatory and multiple organ dysfunction syndrome (MODS). It often leads to pathophysiological damage in gastrointestinal tract, which reacts to progression of sepsis [2]. Gastrointestinal tract is the first involved and the last recovered organ in severe infection, which results in systemic spread and overlapping infection of toxicity and bacteria. It is not only the target organ of MODS, but also promotes failure of other organs in MODS [3, 4].

Enternal nutrition (EN) is a way supplying energy for patients, which not only protects gastrointestinal tract by maintaining mucous membrane completeness, decreasing permeability and increasing blood supply, but also improves prognosis, shortens in-hospital time and reduces complications [5–7]. However, blood supply of gastrointestinal is not sufficient in SS patients, which also induces intestinal intolerance, even non-occlusive mesenteric ischemia and non-occlusive bowel necrosis, after enternal nutrition [8–10]. In clinical practice, the EN of SS patients with early use of vasoactive drugs is still controversial[11–13]. In the NUTRIREA-2 trial, EN and parenteral nutrition (PN) were carried out for shock patients who needed invasive mechanical ventilation and vasopressor drugs. The study found that EN could not reduce the risk of mortality and secondary infection for adult critically ill patients in shock state, but would increase digestive system complications [14]. However, recent studies have shown that EN is safe and tolerable for patients with hemodynamic instability under the maintenance of a certain dose of vasoactive drugs [11, 15, 16].

Thus, this study aimed to explore the effect of NE dose on EN tolerance and prognosis in SS patients.

## Methods and materials

### Baseline characteristics

Totally 150 patients diagnosed as SS from December 2020 to July 2022 in ICU of Shiyan People’s Hospital were included in this retrospective analysis. Patients were divided into tolerance group (n = 97) and intolerance group (n = 53) according to whether EN was tolerated or not.

Patients meeting the following criteria were included: (I) meet the diagnostic criteria of sepsis and SS [17]; (II) age ≥ 18 years old; (III) with more than 24 h overlapping time of norepinephrine and EN; (IV) start EN after enough fluid supplementation and reducing of norepinephrine.

Patients meeting the following criteria were excluded: (I) intestinal obstruction; (II) gastrointestinal bleeding (including hemorrhage in vomitus, residual contents in stomach and feces); (III) severe abdominal infection; (IV) acute pancreatitis; (V) diffuse peritonitis; (VI) gaslrointestinal perforation; (VII) contradiction of EN.

The ethical approval for this study was waived by The ethical committee of Shiyan People’s Hospital (number: syrmyy2022-032) due to its retrospective nature. Informed consent was obtained from all the study subjects before enrollment. This study was performed in accordance with Declaration of Helsinki.

### Methods

Antibiotics, mechanical ventilation, fluid resuscitation, water electrolyte regulation and other treatments in accordance with the international guidelines for management of septic and sepsis shock (2021 Edition) [17] were routinely performed after admission. Patients with SS diagnosed and treated in accordance with the guidelines in ICU [17], who used nasogastric tubes to implement EN when the dosage of vasoactive drugs was reduced, were included in the study. If the patients interrupted EN due to feeding intolerance symptoms in gastrointestinal tract during this period, and were included in the study again when the interruption time exceeded 24 h, they were considered as new cases. The basic information of patients with septic shock, primary disease, invasive mechanical ventilation, CRRT, sedative drugs, cardiotonic, gastrointestinal motility drugs and vasoactive drugs, feeding intolerance index, and prognostic indicators were recorded.

### Study indexes

(I) baseline characteristics: gender, age, weight, body mass index (BMI), scores of acute physiology and chronic health evaluation II system (APACHE II), comorbidity, time in-hospital, prognosis. (II) clinical indexes: mean arterial pressure (MAP), time of mechanical ventilation (MV), norepinephrine dose at the time of starting EN, using of sedative drug, gastrointestinal motility drugs and cardiotonic drugs. (III) EN indexes: timing of starting EN (from the time of patients with sufficient fluid infusion and use of NE), speed of EN infusion, calorie of EN per day, EN target percent. (IV) gastrointestinal intolerance indexes: residual gastric volume > 250 ml, vomiting, aspiration, gastrointestinal bleeding, blood lactic acid (BLA).

### Statistical analysis

All the data collected in this study were analyzed using SPSS 26.0 software. Normally distributed measurement data were expressed as mean ± standard deviation (SD), while non-normally distributed measurement data were expressed as median (interquartile range), and the comparisons were examined by Student-t test and Mann-Whitney test (non parametric distribution). The categorical data were expressed as n (%), and the differences between the two groups were examined by chi-square analysis or Fisher’s exact test. P < 0.05 was considered statistically significant.

## Results

### Baseline characteristics

A total of 150 patients were included in the study, including 80 male patients (53.33%) and 70 female patients (46.67%), with an average age of 66.7 ± 12.7 years. The mean APACHE II score, MAP and BLA concentration were 21.35 ± 5.44, 74.41 ± 8.83 mmHg and 1.74 ± 0.67 mmol. There were 51 (52.58%) male and 46 (47.42%) female patients with a median age of 66.4 ± 12.8 years old in tolerance group. There were 29 (54.72%) male and 24 female (45.28%) patients with a median age 67.3 ± 12.5 years old in intolerance group. The weight and BMI were significantly higher in intolerance group than those of tolerance group (both P < 0.001). It revealed that obesity patients were more likely to be intolerant with EN. There were 18 (18.57%) patients with chronic kidney disease, 16 (16.49%) with heart failure, 20 (20.62%) with diabetes and 16 (16.49%) with hypertension in tolerance group, respectively. Corresponding number of patients in intolerance group were 17 (32.08%), 13 (24.53%), 12 (22.64%) and 12 (22.64%), respectively. There was no significant difference of comorbidity rate between two groups (all P > 0.05). Before the overlapping of EN and norepinephrine, the number of patients receiving gastrointestinal motility drugs in the intolerance group was significantly higher than that in the tolerance group (58.49% vs. 20.62%, P < 0.001). Besides, there was no significant difference of APACHE II score, MAP, BLA, using of mechanical ventilation, implementation of continuous renal replacement therapy, using of sedative drugs, using of cardiac agents and number of patient receiving more than 2 cardioactive drugs between these two groups (all P > 0.05) (Table 1).

**Table 1:** Baseline characteristics of sepsis patients included

Variables	All patients (n = 150)	Tolerance group (n = 97)	Intolerance group (n = 53)	P-value
Sex (male, %)	80 (53.33%)	51 (52.58%)	29 (54.72%)	0.802
Age (year)	66.7 ± 12.7	66.4 ± 12.8	67.3 ± 12.5	0.669
Weight (kg)	68.6 ± 11.2	63.0 ± 8.4	79.0 ± 7.9	< 0.001
Body mass index	25.8 ± 4.4	24.1 ± 3.4	28.9 ± 4.5	< 0.001
Comorbidity (n, %)				
Chronic kidney disease (n, %)	35 (23.33%)	18 (18.57%)	17 (32.08%)	0.061
Heart failure (n, %)	29 (19.33%)	16 (16.49%)	13 (24.53%)	0.234
Diabetes (n, %)	32 (21.33%)	20 (20.62%)	12 (22.64%)	0.773
Hypertension (n, %)	28 (19.33%)	16 (16.49%)	12 (22.64%)	0.356
Before overlapping of EN and NE				
APACHE II score	21.35 ± 5.44	21.07 ± 5.49	21.85 ± 5.37	0.366
Mean arterial pressure (mmHg)	74.41 ± 8.83	73.47 ± 9.31	76.15 ± 7.66	0.683
Blood lactic acid (mmol)	1.74 ± 0.67	1.70 ± 0.71	1.82 ± 0.59	0.311
Mechanical ventilation (%)	138 (92.00%)	91 (93.81%)	48 (90.57%)	0.688
Time of mechanical ventilation (day)	8.7 ± 2.7	8.5 ± 2.8	9.2 ± 2.5	0.088
CRRT (n, %)	94 (62.67%)	66 (68.04%)	28 (52.83%)	0.066
Sedative drugs (n, %)	124 (82.67%)	77 (79.38%)	47 (88.70%)	0.150
Gastrointestinal motility drugs (n, %)	51 (34.00%)	20 (20.62%)	31 (58.49%)	< 0.001
Cardiac agents (n, %)	32 (21.33%)	18 (18.56%)	14 (26.42%)	0.261
Two or more cardioactive drugs (n, %)	38 (25.33%)	20 (20.62%)	18 (33.96%)	0.072

**Abbreviations**: EN: enternal nutrition; NE: norepinephrine; APACHE: Acute Physiology and Chronic Health Evaluation; MAP: mean arterial pressure; CRRT: continuous renal replacement therapy

### Gastrointestinal intolerance indexes during overlapping of EN and norepinephrine

Patients in tolerance group had significantly less residual volume in gastric than that of intolerance group (188.00 ± 52.32 vs. 247.83 ± 34.95 ml, P < 0.001). The rate of residual volume in gastric > 250ml (9.28% vs. 37.74%, P < 0.001), vomiting (15.46% vs. 35.85%, P = 0.004) and aspiration(16.49% vs. 33.96%, P = 0.018) were significantly lower in tolerance group than those of intolerance group. The BLA in tolerance group was significantly lower than that of intolerance group (1.84 ± 0.63 vs. 2.90 ± 1.53 mmol/L, P < 0.001). There were significantly more patients with increased BLA (75.47% vs. 30.93%, P < 0.001) and > 2mmol BLA rising (43.40% vs. 8.25%, P < 0.001) in intolerance group than those of tolerance group. Besides, there was no significant difference of intestinal hemorrhage or perforation on image between two group (P > 0.05) (Table 2).

**Table 2:** Comparison of indexes between tolerance and intolerance group during overlapping of EN and NE.

Variables	All patients (n = 150)	Tolerance group (n = 97)	Intolerance group (n = 53)	P-value
Residual volume in stomach > 250ml (n, %)	29 (19.33%)	9 (9.28%)	20 (37.74%)	< 0.001
Residual volume after intolerance (ml, IQR)	209.1 ± 54.9	188.00 ± 52.32	247.83 ± 34.95	< 0.001
Vomiting (n, %)	34 (22.67%)	15 (15.46%)	19 (35.85%)	0.004
Misinhalation (n, %)	34 (22.67%)	16 (16.49%)	18 (33.96%)	0.018
Gastrointestinal bleeding (n, %)	0 (0.00%)	0 (0.00%)	0 (0.00%)	> 0.999
Blood lactic acid (mmol)	2.2 ± 1.2	1.84 ± 0.63	2.90 ± 1.53	< 0.001
Increase of blood lactic acid (n, %)	70 (46.67%)	30 (30.93%)	40 (75.47%)	< 0.001
Increase of blood lactic acid > 2mmol (n, %)	31 (20.67%)	8 (8.25%)	23 (43.40%)	< 0.001

**Abbreviations**: EN: enternal nutrition; NE: norepinephrine; IQR: interquartile range

### Prognosis and indexes at the time of starting EN

Patients in tolerance group had significantly lower time of starting EN (40.97 ± 9.53 h vs. 49.85 ± 11.61 h, P < 0.001), dose of norepinephrine (0.23 ± 0.07 vs. 0.28 ± 0.10 ug/kg/min, P = 0.049), mortality in hospital (18.56% vs. 49.06%, P < 0.001) and mortality in ICU (16.49% vs. 37.74%, P < 0.001) compared with intolerance group. The EN target percent (92.78% vs. 56.60%, P < 0.001) and calorie of EN during overlapping period (20.22 ± 5.99 vs. 16.21 ± 2.52 kcal/kg/day, P < 0.001) in tolerance group were significantly higher than those of intolerance group. There was no significant difference of overlapping time, speed of norepinephrine infusion, EN energy density, time in-hospital and time in ICU between two groups (all P > 0.05) (Table 3).

**Table 3:** Comparison of indexes between tolerance and intolerance groups during EN and prognoses of these groups

Variables	All patients (n = 150)	Tolerance group (n = 97)	Intolerance group (n = 53)	P-value
Starting of EN from diagnosis (hour)	44.1 ± 11.1	41.0 ± 9.5	49.85 ± 11.61	< 0.001
Overlapping of EN and NE (hour)	83.1 ± 13.1	81.1 ± 18.6	86.9 ± 16.7	0.060
Speed of EN infusion (ml/h)	23.9 ± 7.6	24.7 ± 7.7	22.3 ± 7.4	0.066
EN target percent (n, %)	120 (80.00%)	90 (92.78%)	30 (56.60%)	< 0.001
Calorie of EN during overlapping period (kcal/kg/day,)	18.8 ± 5.4	20.22 ± 5.99	16.21 ± 2.52	< 0.001
Energy density of EN	1.6 ± 0.4	1.6 ± 0.4	1.7 ± 0.4	0.138
Dose of norepinephrine (ug/kg/min)	0.24 ± 0.09	0.23 ± 0.07	0.28 ± 0.10	0.049
EN by stomach tube (n, %)	139 (92.67%)	91 (93.81%)	48 (90.57%)	0.466
Mortality in hospital (n, %)	44 (29.33%)	18 (18.56%)	26 (49.06%)	< 0.001
Mortality in intensive care unit (n, %)	36 (24.00%)	16 (16.49%)	20 (37.74%)	< 0.001
Time of in hospital (days)	17.1 ± 5.2	16.5 ± 4.4	18.2 ± 6.2	0.089
Time of intensive care unit (days)	16.9 ± 4.2	16.4 ± 3.9	17.9 ± 4.3	0.053

**Abbreviations**: EN: enternal nutrition; NE: norepinephrine; IQR: interquartile range

### NE and EN

**Fig. 1:**
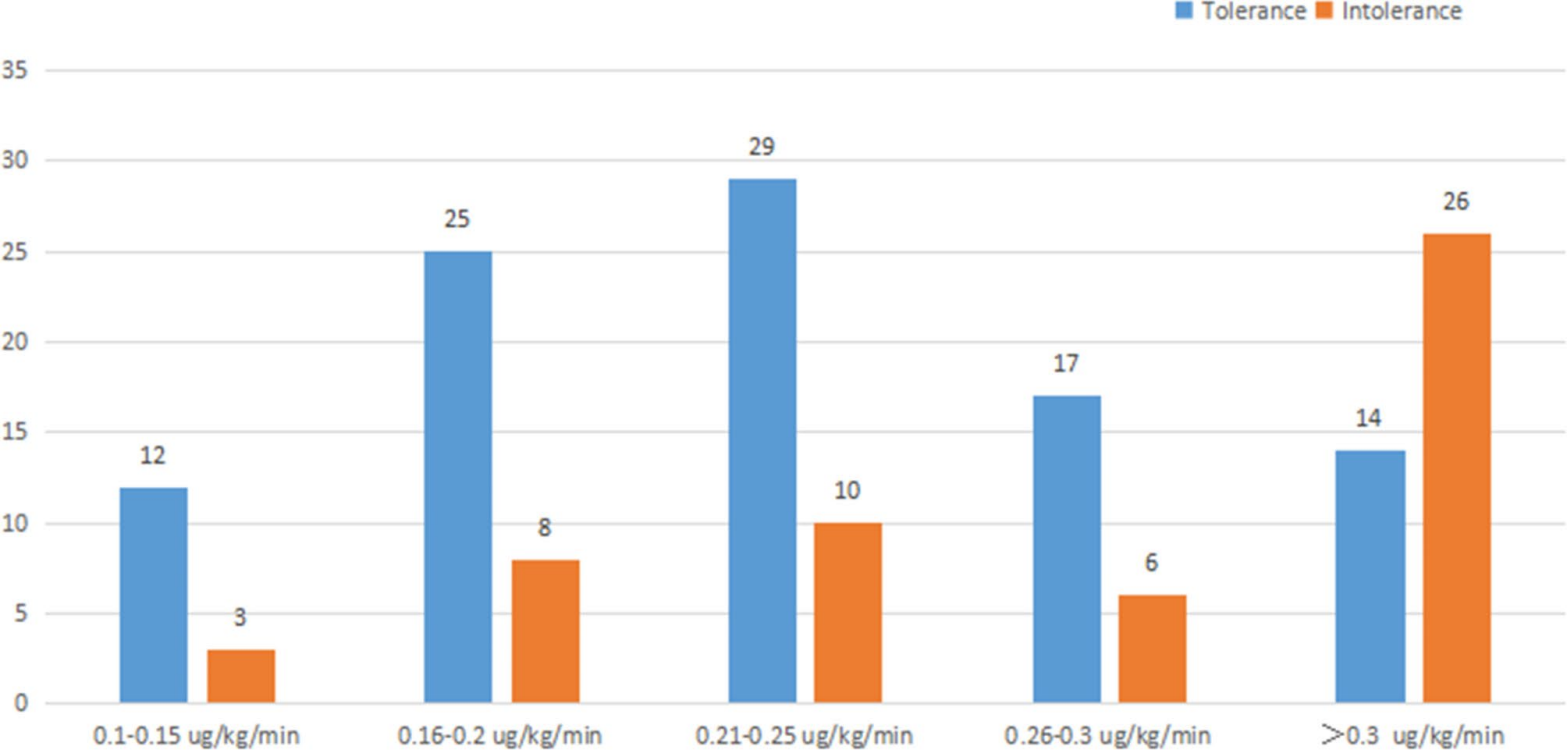
The distribution of patients with different norepinephrine dose in two groups

**Fig. 2:**
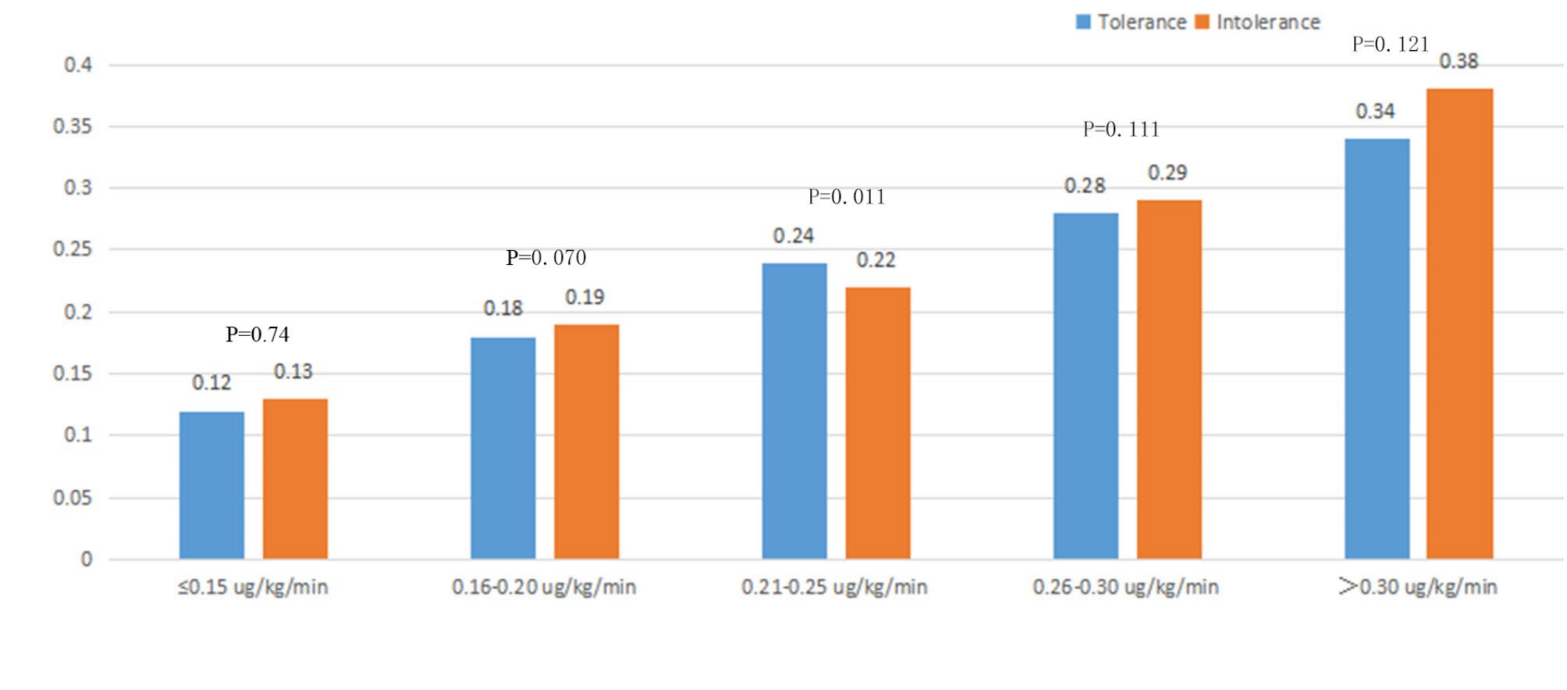
Comparison of NE dose between two groups

The frequency distribution of different NE doses between the tolerance group and the intolerance group was shown in Fig. 1. It was shown that when the NE dose was less than 0.25ug/kg/min, 67% of patients tolerated EN, and when the dose of NE was more than 0.3ug/kg/min, the number of patients who tolerated EN decreased significantly. There was a significant difference between the two groups in the median dose of NE (0.24 vs. 0.22 ug/kg/min, P = 0.011) (Fig. 2).

## Discussion

Septic shock is an intricate problem in the field of acute and critical medicine. There are about 189 of every 100,000 hospital sepsis patients dying of this disease. The hypermetabolic state caused by severe stress reaction and insufficient energy intake is easy to lead to malnutrition, and will affect the treatment and prognosis of these patients [18–20]. Although SS maintains the stability of hemodynamics through adequate fluid infusion and vasoactive drugs, there are still risks of gastrointestinal ischemia, hypoxia and hypoperfusion, etc. [15].

As one of the key process in treatment of SS, EN plays a vital role in improving intestinal mucous atrophy and villus damage, inhibiting enterogenous infection, and improving immunity [6, 21]. However, EN also has the risk of aggravating intestinal ischemia and hypoxia. At present, there is still controversial among various authoritative guidelines on the timing of EN initiation in SS patients with unstable hemodynamics. The American Society for Parenteral and Enteral Nutrition (ASPEN) proposed the Nutrition Guidelines for Critically Ill Patients (2016 Edition) [22], which recommended that SS patients with unstable hemodynamics could suspend using of EN, and patients who successfully resuscitate or begin to withdraw vasoactive drugs could receive EN. However, the guidelines for SS [17] held different views, which proposed that EN should be given as early as tolerance of patients. Merchant applied EN to 120 patients with SS who received vasoactive drugs [11]. It showed that obese patients were more likely to be intolerant with EN when SS occurred, and 62% of patients were tolerant of EN without intestinal ischemia, which was consistent with the present study. It was also found safe and tolerable to start EN within 48 h if SS patients received norepinephrine with an equivalent amount less than 0.14 mg/kg/min after adequate fluid resuscitation. This study found that EN was started within 48 h and when NE dose was ≤ 0.25 mg/kg/min, 67% of patients tolerated EN. Mancel [16] et al. found that 74.9% of patients could tolerate EN at the same time of receiving vasoactive drugs, and the maximum dose of norepinephrine tolerated was ≤ 12.5ug/min. The most commonly complication was BLA increase (30.6%) among these patients. This study also found that gastrointestinal intolerance event was mainly increased BLA (40%). Hubangchuan [15] et al. found that EN could be tolerated in critical patients receiving stable dose of vasoactive drugs to maintain hemodynamic stability, if norepinephrine dose was less than 0.2ug/kg/min. SS Patients are in critical condition and progress rapidly. Timely and effective treatment could improve the prognosis and reduce the occurrence of complications. EN could promote the recovery of intestinal function, which was more in line with the physiological state of the body, showing that earlier EN implementation could result in better the prognosis among these patients [23].

There were also several limitations in this study. Firstly, this were unavoidable biases in this analysis due to its retrospective nature. Secondly, this is a single center analysis with small sample size. Thus, all results in present study should be interpreted cautiously. Prospective study with large sample should be conducted in the future.

## Conclusion

In conclusion, SS patients should be comprehensively evaluated according to their condition. Obese patients are more prone to EN intolerance, and those who can tolerate EN should be implemented as soon as possible. The use dose of NE is significantly related to EN tolerance. When the use dose is low, EN tolerance is greater.

## Data Availability

The datasets generated and analyzed during the current study are available from the corresponding author on reasonable request.

## Abbreviations

BMI: Body mass index
BLA: Blood lactic acid
EN: Enteral nutrition
ICU: Intensive care unit
MODS: Multiple organ dysfunction syndrome
MAP: Mean arterial pressure
MV: Time of mechanical ventilation
PN: Parenteral nutrition
SS: Septic shock
SD: Standard deviation
